# Dynamic proteomic analysis of *Aedes aegypti* Aag-2 cells infected with Mayaro virus

**DOI:** 10.1186/s13071-020-04167-2

**Published:** 2020-06-10

**Authors:** Anna Fernanda Vasconcellos, Samuel Coelho Mandacaru, Athos Silva de Oliveira, Wagner Fontes, Reynaldo Magalhães Melo, Marcelo Valle de Sousa, Renato Oliveira Resende, Sébastien Charneau

**Affiliations:** 1 Laboratory of Protein Chemistry and Biochemistry, Department of Cell Biology, Institute of Biology, University of Brasilia, Brasilia, DF 70910-900 Brazil; 2 Laboratory of Virology, Department of Cell Biology, Institute of Biology, University of Brasilia, Brasilia, DF 70910-900 Brazil

**Keywords:** MAYV, Mosquito, Infection, Virus-vector interaction, Semi-quantitative proteomics, Differentially expressed proteins

## Abstract

**Background:**

Mayaro virus (MAYV) is responsible for a mosquito-borne tropical disease with clinical symptoms similar to dengue or chikungunya virus fevers. In addition to the recent territorial expansion of MAYV, this virus may be responsible for an increasing number of outbreaks. Currently, no vaccine is available. *Aedes aegypti* is promiscuous in its viral transmission and thus an interesting model to understand MAYV-vector interactions. While the life-cycle of MAYV is known, the mechanisms by which this arbovirus affects mosquito host cells are not clearly understood.

**Methods:**

After defining the best conditions for cell culture harvesting using the highest virus titer, *Ae. aegypti* Aag-2 cells were infected with a Brazilian MAYV isolate at a MOI of 1 in order to perform a comparative proteomic analysis of MAYV-infected Aag-2 cells by using a label-free semi-quantitative bottom-up proteomic analysis. Time-course analyses were performed at 12 and 48 h post-infection (hpi). After spectrum alignment between the triplicates of each time point and changes of the relative abundance level calculation, the identified proteins were annotated and using Gene Ontology database and protein pathways were annotated using the Kyoto Encyclopedia of Genes and Genomes.

**Results:**

After three reproducible biological replicates, the total proteome analysis allowed for the identification of 5330 peptides and the mapping of 459, 376 and 251 protein groups, at time 0, 12 hpi and 48 hpi, respectively. A total of 161 mosquito proteins were found to be differentially abundant during the time-course, mostly related to host cell processes, including redox metabolism, translation, energy metabolism, and host cell defense. MAYV infection also increased host protein expression implicated in viral replication.

**Conclusions:**

To our knowledge, this first proteomic time-course analysis of MAYV-infected mosquito cells sheds light on the molecular basis of the viral infection process and host cell response during the first 48 hpi. Our data highlight several mosquito proteins modulated by the virus, revealing that MAYV manipulates mosquito cell metabolism for its propagation.
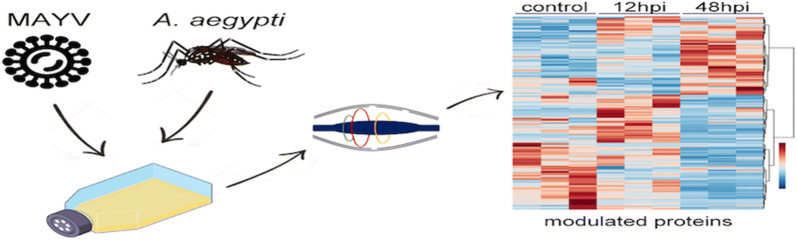

## Background

Mayaro virus (MAYV) is an arbovirus (arthropod-borne virus) that causes febrile illness and arthralgia in humans [1]. The virus belongs to the genus *Alphavirus*, family *Togaviridae*, sharing biological and medical aspects with other important arboviruses, such as chikungunya virus (CHIKV) [2]. MAYV is an emerging and not well recognized virus, so MAYV fever has been frequently misdiagnosed as other mosquito-borne diseases, including dengue virus (DENV) fever, due to their similar clinical symptoms [3]. The majority of countries that have reported autochthonous cases of MAYV are in Latin America [4].

MAYV has a positive single-stranded RNA genome of about 11.2 kb that encodes two polyproteins [5]. Both of them are cleaved by host and viral proteases in the cytoplasm, originating mature non-structural (nsP1, nsP2, nsP3 and nsP4) and structural (CP, E3, E2, 6k and E1) proteins [6]. The non-structural proteins are directly translated from the genomic RNA and, in addition to other functions, catalyze viral RNA synthesis. The structural proteins are translated from a subgenomic RNA and assemble to form the virus particles [7].

Despite its restricted area of distribution, MAYV can be transmitted to humans by mosquitoes of at least four different genera, having a great potential for expansion. *Haemagogus* spp. are the main vectors, but transmission has also been reported from *Culex* spp., *Sabethe* spp. and *Aedes* spp. [4, 5]. Understanding the virus-vector interactions is one of the ways to develop strategies for virus control. Viruses are intracellular parasites with small genomes that hijack and manipulate the host cell machinery for their own replication [8, 9]. In this context, host proteins perform important roles during the virus cycle and are key factors in understanding the steps involved in virus infection and therefore in developing methods in halting virus replication.

*Aedes aegypti* is well adapted to urban domestic habitats and has a strong human-feeding preference. Moreover, its widespread colonization and distribution in the tropics, has meant that this mosquito species has become highly adapted to urban tropical areas [10]. *Aedes aegypti* is very promiscuous concerning viral transmission, making it an interesting research model to understand virus-vector interactions [11, 12]. The availability of the *Ae. aegypti* Aag-2 cell line also facilitates the establishment of infected cell cultures under controlled environmental conditions. In this study, we evaluated the proteome of Aag-2 cells infected with MAYV by using label-free mass spectrometry. As a result, mosquito proteins that are important for MAYV replication have been identified, as well as proteins that may act as antiviral agents inhibiting virus replication.

## Methods

### Cells and virus

Vero cells (African green monkey kidney epithelial cells) were grown in high-glucose Dulbecco’s modified Eagleʼs medium (DMEM; Sigma-Aldrich, St. Louis, Missouri, USA) supplemented with 10% fetal bovine serum (FBS), and with 100 U/ml penicillin/streptomycin at 37 °C under 5% CO_2_. *Aedes albopictus* C6/36 cells were grown in TC-100 medium (Vitrocell Embriolife, Campinas, SP, Brazil) supplemented with 10% FBS at 28 °C. *Aedes aegypti* Aag-2 cells (provided by Gorben Pijlman from Wageningen University and Research, Wageningen, Netherlands) were grown in Schneider’s medium (Sigma-Aldrich) supplemented with 10% FBS, and with 100 U/ml penicillin/streptomycin at 28 °C. Cell passages were performed every 3–4 days. The MAYV isolate (strain Campos-RJ, Brazil) was supplied by Instituto Oswaldo Cruz (Rio de Janeiro, Brazil). The MAYV isolate was first inoculated in a mammalian Vero cell monolayer culture, which is commonly susceptible and permissive to arboviruses and presents clear cytopathic effects (CPE) during the onset of virus replication.

### Mayaro virus propagation

Once received, the MAYV isolate (in cell culture medium) was inoculated in Vero cells grown in a T25 flask. The supernatant was collected 3 days post-infection (dpi) following the onset of CPE. Virus replication was also confirmed by RT-PCR after RNA extraction of Vero cells with Trizol (Thermo Fisher Scientific, Waltham, MA, USA). For cDNA synthesis, SuperScript IV reverse transcriptase (Thermo Fisher Scientific) was used. PCR was then performed with Platinum Taq DNA polymerase (Thermo Fisher Scientific) with the degenerate primers (5′-GCR GCY YCG ACA GTG ACA GCY AT-3′ and 5′-TGC ATG YGC TTT CGG TGC RC-3′), which amplified the E3-E2 genes (about 1.5 kb). All procedures followed the manufacturers’ recommendations. To increase the virus titer for further infection experiments using Aag-2 cells, *Ae. albopictus* C6/36 cells were infected with Vero-derived culture supernatant. These mosquito cells have a defective antiviral RNA interference response, which is advantageous for arbovirus propagation [13]. C6/36 supernatants were collected for virus titration and further use.

### Virus titration by end-point dilution assay

Confluent Vero cells grown in T25 flasks were detached with 1 ml trypsin-EDTA (Gibco, Carlsbad, CA, USA) and then diluted in 14 ml supplemented DMEM. The Vero cells, in suspension, were incubated with serial dilutions (in supplemented DMEM) of virus stocks in a 1:1 ratio (90 µl cell suspension: 90 µl virus dilution). A volume of 10 µl per well was plated 6-fold (per dilution) in 60 well Terasaki plates (Greiner Bio-One, Kremsmünster, Austria). Finally, the end-point dilution of infected cells was scored based on CPE visualized by light microscopy at 3–5 dpi.

### Growth kinetics in Aag-2 cells

To explore the proteomic changes of Aag-2 cells upon infection with MAYV, we first determined the best conditions for cell culture harvesting according to the highest virus titer. Aag-2 cells were infected in 6-well plates at MOI 0.1 and 1 in biological triplicates. After 90 min of adsorption, the supernatant was carefully removed and replaced with fresh Schneider’s medium. The final MAYV-infected supernatant was titrated at 24, 48 and 72 hpi by end-point dilution assay (EPDA), and a virus growth curve was determined.

### Sample preparation for mass spectrometry

Approximately 2 × 10^6^ Aag-2 cells were seeded in T25 flasks and infected with MAYV 16 h later at MOI 1, in triplicate. Cells, including mock-infected cells with Schneider’s medium, were harvested at 12 hpi and 48 hpi. Samples were reduced with 10 mM dithiothreitol (DTT) in 0.25 M Tris pH 8.6 for 1 h at 52 °C and then alkylated with 50 mM iodoacetamide for 1 h at 25 °C. After digestion with trypsin (Promega, Madison, WI, USA) at a ratio 1:50 (enzyme:substrate), the samples were desalted through a pipette tip packed with a C18 membrane (PerSeptive Biosystems, Framingham, USA) and dried.

### Mass spectrometry

An Orbitrap Elite^TM^ hybrid ion trap-orbitrap mass spectrometer (Thermo Fisher Scientific) was used for LC-MS/MS analysis. Samples were resuspended in 0.1% (v/v) formic acid and loaded into a nano-UPLC-Dionex 3000 system (Thermo Fisher Scientific) equipped with a C18 trap type column (100 μm × 3 cm with particles of 5 μm/100 Å) and a C18 analytical column (75 μm × 35 cm with particles of 3 μm/100 Å). Peptides were eluted from the analytical column with a gradient of 2% to 35% of solvent B (0.1% (v/v) formic acid in acetonitrile) for 15 min and loaded directly into the mass spectrometer under electrospray ionization. Molecular mass spectra were acquired using the data-dependent acquisition (DDA) mode controlled by Xcalibur 2.0 software (Thermo Fisher Scientific). The DDA acquisition cycle comprised the range of 350–1650 m/z and a resolution of 120,000 for MS1. The twenty most abundant precursor ions were fragmented by collision-induced dissociation (CID) during 90 s of dynamic exclusion and collision energy normalized at 35%.

### Label-free protein quantification and identification

For all biological replicates, quantification was performed using the Progenesis QI for proteomics software (version 1.0; Nonlinear Dynamics, Newcastle upon Tyne, UK). The spectra were aligned and quantified based on high mass accuracy MS1 events. Relative changes in abundance level were calculated for each detected MS1 event by comparing the peak areas calculated using extracted ion chromatogram. The changes were compared among LC-MS/MS runs according to the experimental design for replicates and conditions. No internal reference peaks were used. Fragment ion scans were exported as Mascot generic files (mgf) and searched with PEAKS Studio 7.0 (Bioinformatics Solutions Inc., Ontario, Canada) using the compiled UniProt “*Ae. aegypti* + MAYV” protein database (25,023 sequences accessed on 2 December 2018). The parameters used were: 10 ppm peptide mass tolerance; 0.5 Da fragment mass tolerance; and two missed cleavages allowed. Methionine oxidations and acetylation of protein N-termini were specified as variable modifications, while carbamidomethylation of cysteine was specified as a fixed modification. Protein identities were assigned if at least two unique peptides were matched using a false discovery rate (FDR) of < 1%. Identifications were re-imported into Progenesis QI, for protein quantification. Some identified peptides could be responsible for the identification of more than one protein, called conflicting peptides. These were detected and the most probable protein identification for each peptide was manually chosen according to the protein score, resolving the conflicts. Peptides still in conflict after manual validation were excluded from protein quantification.

### Statistical analysis and biological context

All statistical analysis was performed on protein relative abundance data obtained through Progenesis QI software (Nonlinear Dynamics; http://www.nonlinear.com/progenesis/qi). After normalization and transformation, data were analyzed by principal components analysis (PCA) provided in the R programming environment [14], through the *FactoMineR* package [15]. PCA was performed at both peptide and protein levels to evaluate whether the grouping among replicates was more evident than the grouping of conditions, as well as to detect for the possible presence of outliers. Three technical replicates were carried out for each sample preparation to confirm reproducibility. Proteins were considered significantly regulated among conditions based on ANOVA with *P*-values *P* < 0.05. After being identified, the protein sequences were submitted to Gene Ontology annotation by Blast2GO software (BioBam Bioinformatics, Valencia, Spain) and to BlastKOALA in the Kyoto Encyclopedia of Genes and Genomes (KEGG- http://www.kegg.jp/blastkoala/) for functional annotation. Metaboanalyst 4.0 (https://www.metaboanalyst.ca/) was used to normalize and cluster the data, as well as to perform PCA analysis and plot heatmap graphs [16]. To better understand protein dynamics, the samples from the heatmap were clustered using the VSClust online platform (http://computproteomics.bmb.sdu.dk/Apps/VSClust/), a clustering tool based on fuzzy c-means clustering that considers the variance of each feature [17].

## Results and discussion

Despite Mayaro fever outbreaks having been recorded in Brazil, Bolivia and Peru since 1978 [18] and despite the recent emergence of MAYV recombinants that are more adapted to humans in Brazil and Haiti [19], the MAYV infection ability and its molecular pathology remain obscure. A label-free semi-quantitative bottom-up proteomic analysis over-time was performed to see how *Ae. aegypti* Aag-2 cells respond to MAYV infection during different time points of infection.

### Infection of *Aedes aegypti* Aag-2 cells with MAYV

**Fig. 1:**
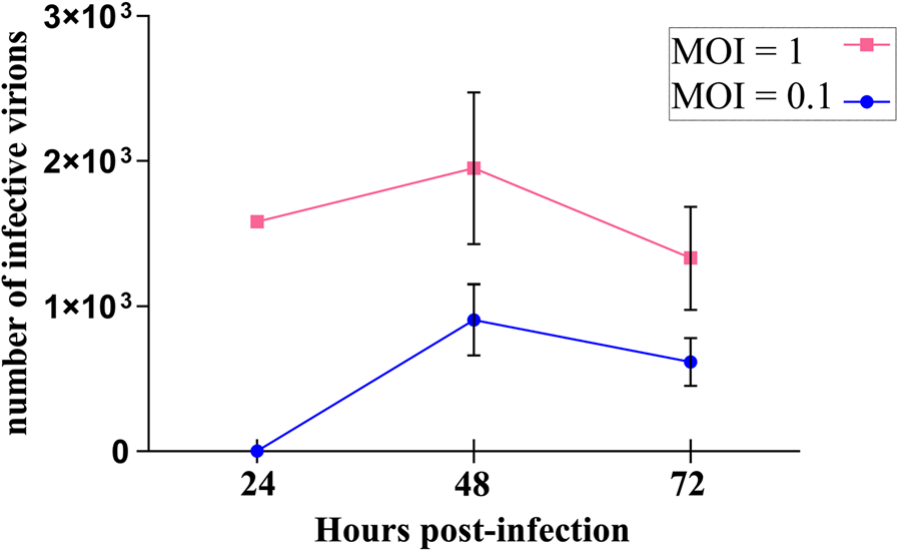
MAYV growth kinetics in Aag-2 cells. Aag-2 cells were infected with MAYV at a MOI of 1 and were harvested at the indicated time points. Graphics obtained using GraphPad Prism 6

The highest MAYV titer obtained when infecting Aag-2 cell culture was about 2 × 10^3^ TCID_50_/ml at MOI 1 and 48 hpi (Fig. 1). At this time point (48 hpi), a two-fold change of infectious viruses was observed between MOI 0.1 and 1. Based on these observations, the infection for proteomic analysis was carried out at MOI 1 and cell harvesting was performed at 12 hpi and 48 hpi.

### Dataset analysis

**Fig. 2:**
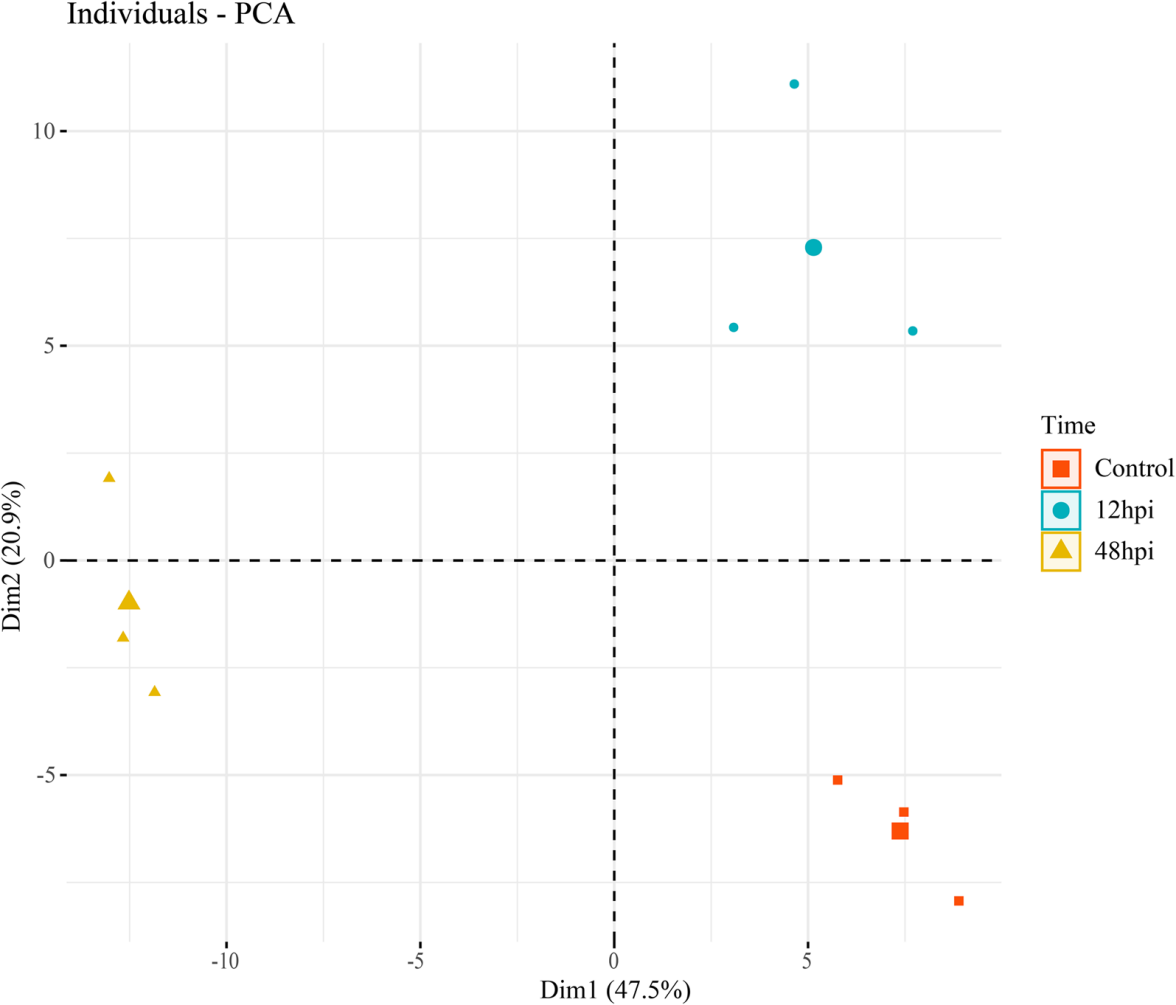
Principal components analysis of the proteomic dataset. Orange squares are triplicate measurements at time 0 (corresponding to uninfected Aag-2 as control), blue dots and yellow triangles are the triplicates at times 12 hpi and 48 hpi, respectively. The larger geometric symbols represent the centroid of each cluster triplicates. Graphics obtained using *factorextra* R Package, R environment

Following mass spectrometry, the PCA revealed that the data clustered more tightly with their replicates rather than experimental conditions (Fig. 2). The variable correlation plot indicates a large number of variables of good quality contributing to the formation of the principal components (Additional file 1: Figure S1).

**Fig. 3:**
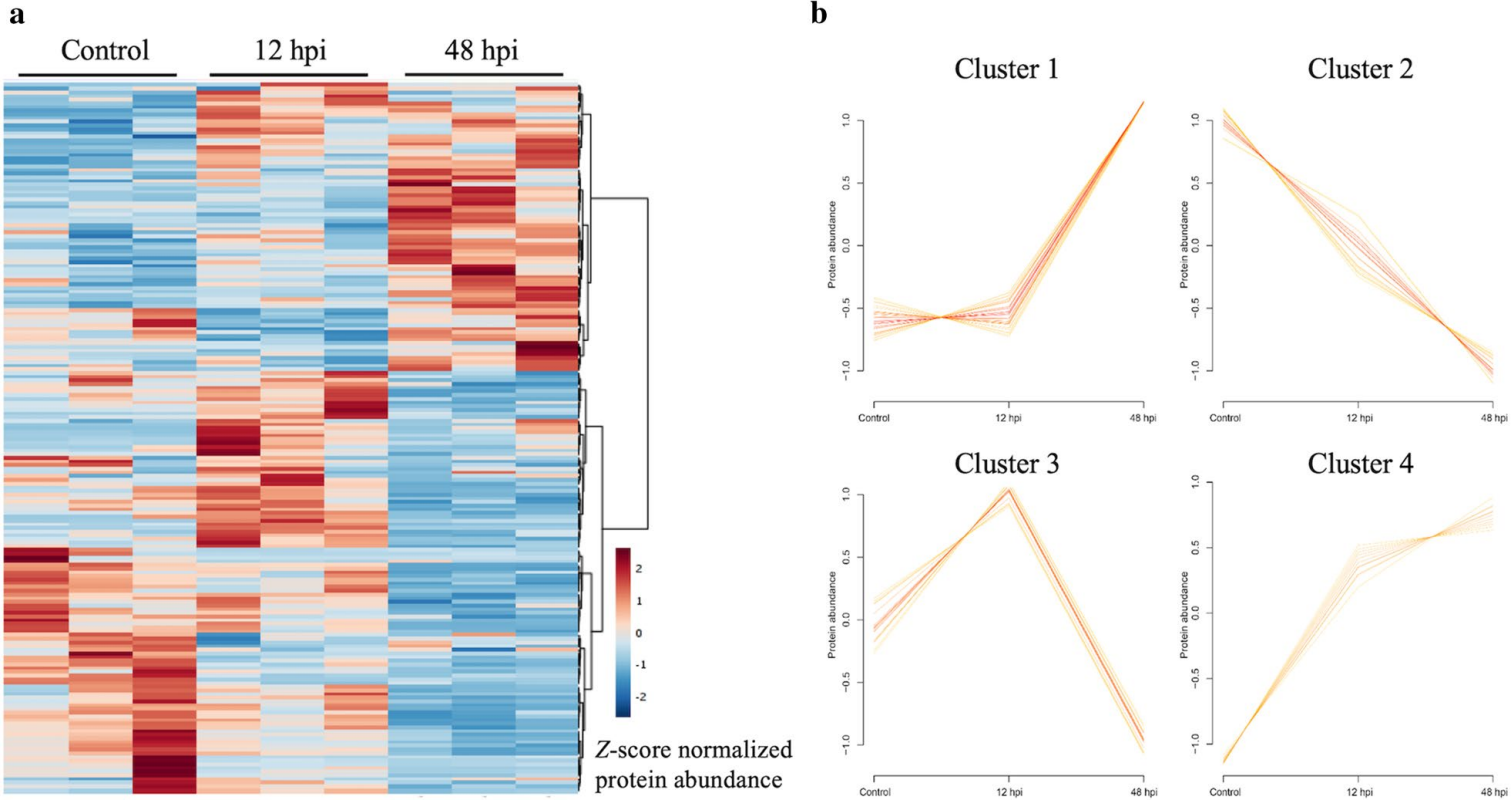
Abundance of all Aag-2 quantified proteins over the different infection time points. **a** Heatmap presenting all Aag-2 quantified proteins. Graphics obtained using MetaboAnalyt 4.0. Proteins hierarchically clustered across all samples as shown on the right. **b** Protein dynamic abundance clusters over condition groups obtained through the VSClust algorithm, where the protein abundance is represented by a log-transformed normalized value

The abundance variation pattern of all identified proteins over the different infection time points was visualized in a heatmap (Fig. 3a) from which four clusters emerged (Fig. 3b and Additional file 2: Table S1). The optimal number of clusters was defined according to the minimum centroid distance of each number of clusters tested (Additional file 3: Figure S2).

The clustering patterns highlighted protein similarity across the groups. Proteins in cluster 1 demonstrated similar abundances in control and 12 hpi, but increased in 48 hpi. A total of 33 proteins were grouped in this cluster. Protein abundance in cluster 2 decreased compared to control at 12 hpi and 48 hpi. This cluster represented 20 proteins. In cluster 3, protein abundance increased at 12 hpi compared to the control and drastically decreased from 12 hpi to 48 hpi, grouping 17 proteins. Cluster 4 demonstrated progressively increased abundance from control to 12 hpi, then to 48 hpi with a slight decrease in slope, representing 11 proteins (Fig. 3b, Additional file 1: Figure S1).

### Global proteomics of MAYV-infected Aag-2 cells

**Fig. 4:**
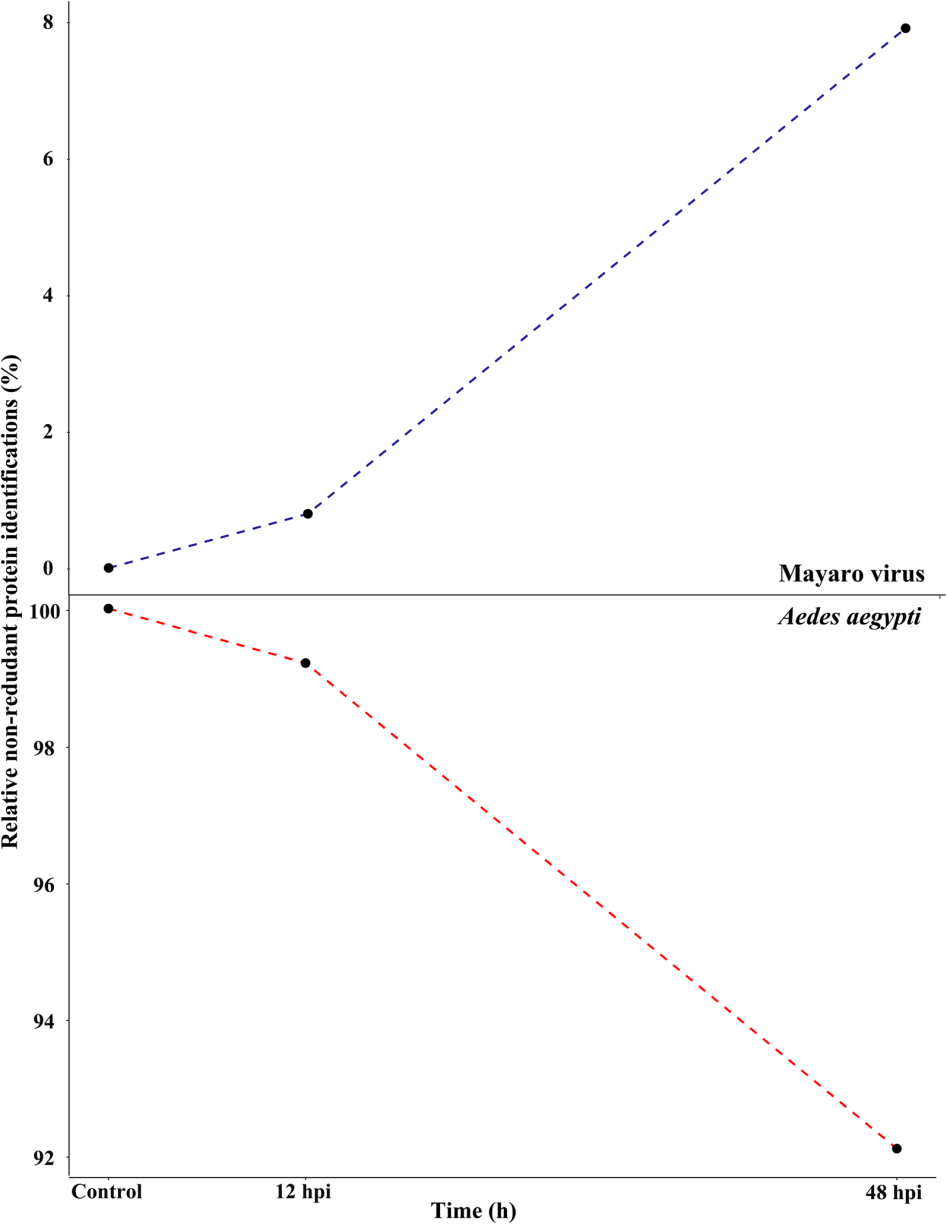
Relative non-redundant protein identifications (%) from MAYV (blue) and *Aedes aegypti* Aag-2 cells (red) over the different infection time points. Graphics obtained using R environment

From three time-course infection experiments, three biological samples were harvested at each time point (0, 12 hpi and 48 hpi). The total proteome analysis allowed the identification of 5330 peptides (Additional file 4: Table S2) providing a total of 564 non-redundant proteins of Aag-2 cells. The analysis yielded the identification of 459, 378 and 253 protein groups, at time 0 (corresponding to uninfected Aag-2 cells, as a control), 12 hpi and 48 hpi, respectively. A full list of the identified proteins is given in Additional file 5: Table S3. While the number of identified *Ae. aegypti* proteins drastically decreased, the number of identified MAYV proteins increased along the 48 h of infection (Fig. 4). During the viral infectious cycle, changes in represented insect cell pathways reflected the onset of the fast viral replication that explain an increase of viral protein abundance. Moreover, part of the infected cells in culture died, and consequently their proteins were progressively released in the culture medium from disrupted cells or degraded by proteolysis, explaining the decrease of the identification amount at the post-infection time points.

### Dynamic proteomics of MAYV-infected Aag-2 cells

A relative analysis of the changes in mosquito protein abundance between the three time points was performed using proteins that could be identified based on having two matching peptides. The abundance of 161 proteins changed significantly over the time course of infection (ANOVA *P*-values < 0.05) (Additional file 6: Table S4**)**. Thus, the changes observed in the Aag-2 cell proteome were likely to be related to MAYV infection effects. Many of the protein changes we observed have been recorded from previous infection experiments with arboviruses [20, 21]. These include heat-shock proteins, ATP synthase and enolase phosphatase e1 (see below).

**Fig. 5:**
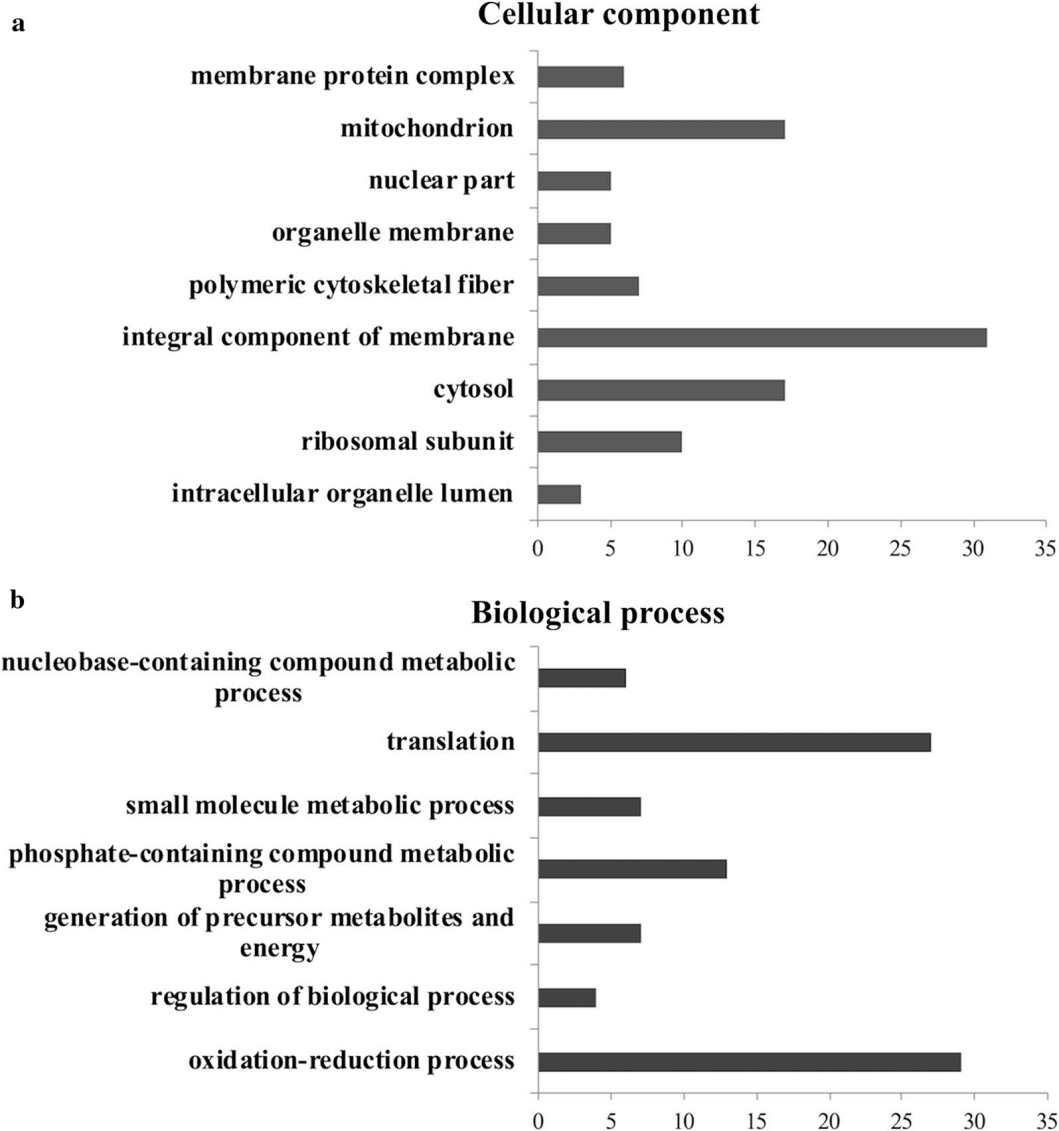
Functional annotation of 161 proteins significantly differentially expressed in response to MAYV infection of Aag-2 cells. Bar charts represent the percentages of proteins associated to GO terms cellular component (**a**) and biological process (**b**), obtained using the software Blast2Go

The Gene Ontology (GO) classifications of the modulated proteins according to cellular distribution and biological process are shown in Fig. 5a, b and listed in Additional file 6: Table S4. A large number of modulated proteins were related to bio-membranes, showing that our sample preparation method was efficient in recovering hydrophobic proteins. Proteins belonging to integral membrane component, organelle membrane and membrane complex categories comprised 31%, 5% and 6% of the 289 proteins identified, respectively. Other proteins were mainly assigned to cytosol or mitochondrion categories (17% each) (Fig. 5a). The modulated proteins were mostly related to redox processes (29%), translation (27%) and other metabolic processes of compounds. The metabolism of phosphate-containing compounds represented 13% of these (Fig. 5b). Therefore, the effect of the infection on the mosquito proteome was mostly associated with response to oxidative stress, homeostasis and protein folding, protein synthesis and ATP production.

Interestingly, *Ae. aegypti* proteins grouped in clusters 1, 3 and 4 (Fig. 3b) showed increased abundance at 12 hpi or 48 hpi against the overall trend of decreasing representation of mosquito proteins as the infection progressed.

Globally, Aag-2 cell proteome change was more clearly identified at 48 hpi than 12 hpi compared to time 0. Given that the time required for replication of a single alphavirus genome is 4 h [20], the mosquito protein abundance variations may be associated with the increasing burden of infection.

### Viral particle internalization

It is well known that arboviruses such as MAYV can infect both mosquito and vertebrate cells, suggesting that the host cell receptors involved with its internalization are shared between these cell types [21]. Besides virus internalization into insect and mammalian cells is mostly dependent on clathrin-mediated endocytosis, other surface receptors have been implicated in viral cell entrance [20]. In this study, prohibitin was upregulated in *Ae. aegypti* cells at 48 hpi (Additional file 6: Table S4). This surface receptor has already been characterized as a mediator of dengue virus serotype 2 entrance into *Ae. albopictus* and *Ae. aegypti* cells [22]. Prohibitin is also a mediator entrance of alphaviral CHIKV particles into mammalian cells from different lineages, even though its precise role is still unknown [23]. Thus, we propose that prohibitin may also mediate MAYV virus internalization in mosquito cells. However, given its ability to infect different cells, we cannot discard the possibility that MAYV may have other receptors and internalization mechanisms.

### Cell response to stress

The heat-shock proteins (HSPs) are a large family of widely abundant and evolutionarily conserved molecular chaperones that act in cellular homeostasis, mainly in response to stress [24]. They act in the correct folding, assembly and traffic of proteins and protein complexes. Upregulation of chaperone expression, in general, can be triggered by different stimuli such as elevated temperatures, nutrient shortages or viral infections [25]. Here, HSP-20 and HSP-60 of Aag-2 cells showed increasing abundance throughout the infection (Additional file 6: Table S4). It was previously shown that, by increasing the incubation temperature of MAYV-infected *Ae. albopictus* C6/36 cells, the expression of HSPs was induced and viral replication was strongly inhibited [26]. More recently, C6/36 cells infected with the alphavirus CHIKV also induced overexpression of HSP-60 [27]. It was hypothesized that these chaperones could be acting in favor of viral replication. However, after knockout of this siRNA-mediated chaperone, C6/36 cells showed an increase in viral titre [27]. Moreover, transcriptomic analysis of *Drosophila* cell culture showed the induction of chaperones when infected with RNA viruses [28]. Altogether, these data point to a conserved antiviral mechanism and corroborate that chaperones are important in the response against MAYV in *Ae. aegypti* cells. Beyond this, one HSC70, the heat-shock protein 70 cognate 5, was also identified as upregulated in Aag-2 cells at 12 hpi with MAYV (Additional file 6: Table S4). The HSC70/HSP90 machinery is an important mediator of the RNA-induced silencing complex (RISC) assembly pathway for the RNA interference (RNAi) antiviral defense mechanism, helping the conformational opening of Argonaute proteins to receive small RNA duplexes in an ATP dependent manner [29]. Since the RNAi machinery is the main antiviral mechanism in invertebrates, the super expression of HSC70 may be a defense mechanism of the infected Aag-2 cells. In fact, *Anopheles gambiae* HSC70B has been implicated in viral replication blocking when infected with the togavirus O’Nyong-Nyong [30].

- Another protein associated with stress response is enolase phosphatase e1 (ENOPH1). Positively regulated throughout MAYV infection (Additional file 6: Table S4), ENOPH1 is an enzyme involved in L-methionine biosynthesis *via* the salvage pathway. This process catalyzes the conversion of methyladenosine back to l-methionine [31], and results in the production of polyamines, small cationic molecules essential for cell development and homeostasis [32]. The overexpression of ENOPH1 has already been found in *Ae. albopictus* cell culture infected with DENV and with CHIKV [27, 33]. However, the CHIKV titer increased in knockout mosquito cell lines of the ENOPH1 gene, implying a function in regulating the stress response and innate immunity of host cells during infection. [27]. Therefore, the importance of the host ENOPH1 factor for viral replication could indicate a higher production of polyamines during infection.

### Energy and intermediate metabolite demand

**Fig. 6:**
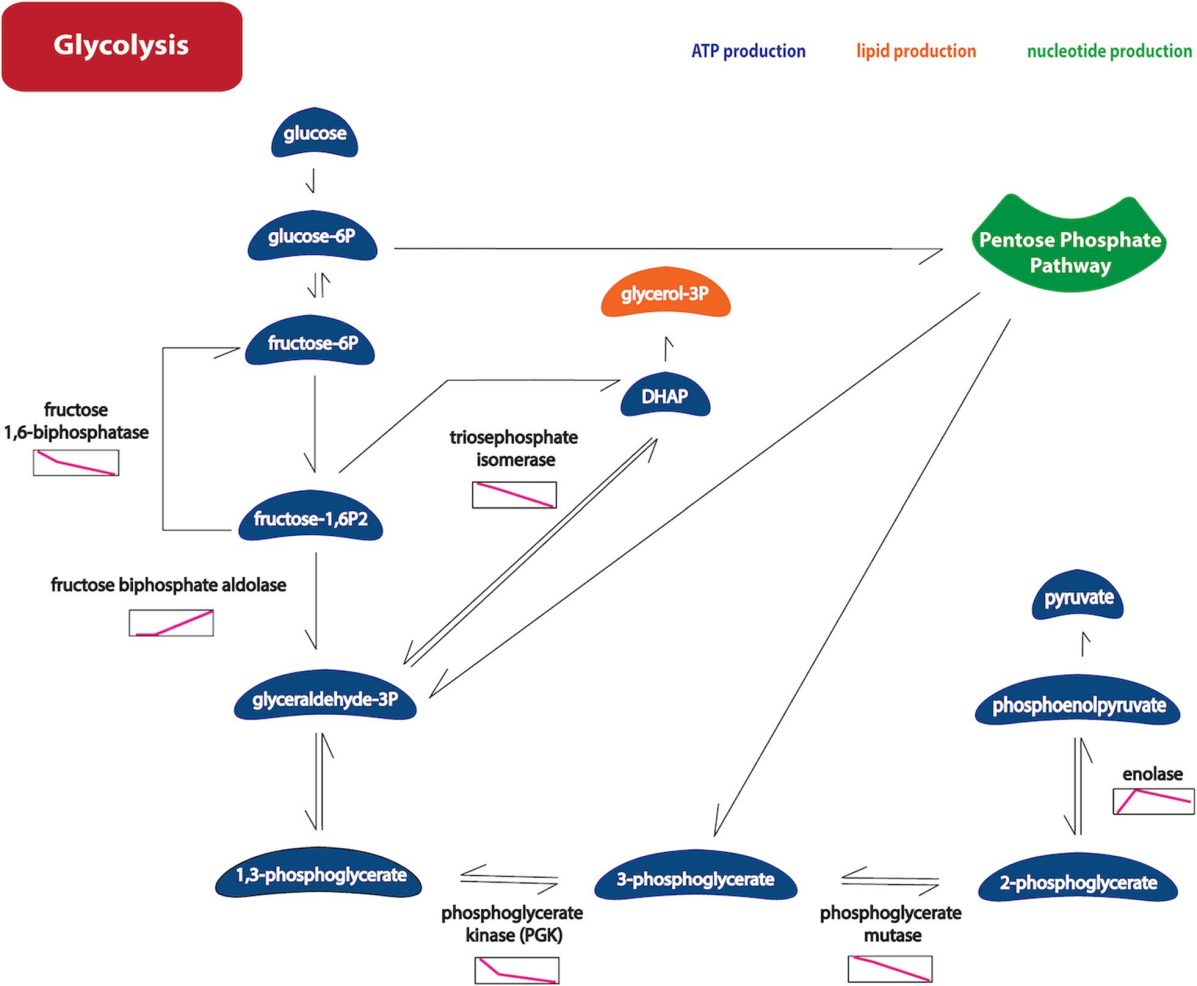
Modulated proteins of glycolysis pathway. The individual identified proteins were placed into KEGG pathways by the use of BLASTKOALA. The graphics near the enzymes show their abundance pattern (Y-axis) among the three times of infection (X-axis): 0 h, 12 hpi and 48 hpi. Default search parameters were used against the “family_eukaryotes” KEGG Genes Database (http://www.kegg.jp/kegg/kegg1.html). Image obtained using R environment and Adobe Illustrator CS6

Both glucose and glutamine have been characterized as carbon sources for human viruses [34]. In this study, nine modulated *Ae. aegypti* enzymes that act in glycolysis were identified (Fig. 6, Additional file 6: Table S4). According to their pattern of protein abundance at 48 hpi with MAYV, increased glycolytic activity was implied, as well as an accumulation of metabolic intermediates. The decreased abundance of fructose 1,6-biphosphatase, phosphoglycerate kinase and phosphoglycerate mutase associated with an increased abundance of fructose-biphosphate aldolase and triosephosphate isomerase could have favored glyceraldehyde-3-phosphate accumulation. This glycolytic intermediate can be easily isomerized to dihydroxyacetone phosphate (DHAP), which is also involved in lipid production. Moreover, the pentose phosphate pathway, required for the synthesis of ribonucleotides and the production of the reducing agent NADPH, branches glyceraldehyde-3-phosphate from glycolysis. However, the increased abundance of the key glycolytic enzyme enolase may also be associated with the increase of glycolytic activity. The alterations in the proteins involved in the glycolytic pathway are probably not only due to rapid ATP production due to the high energy demand of the MAYV infectious cycle. Alphaviruses replicate their genome in association with cellular membranes and the budding of new virions can also occur in internal vesicles [35]. Therefore, nucleotide and lipid production could also have been activated by MAYV to facilitate its replication in Aag-2 cells. Furthermore, mitochondrial membrane disruption, with a consequent decrease in ATP levels, has been observed in influenza virus-infected cells, followed by glycolysis activation to reset energy levels [36]. For MAYV, another evidence of this energy demand is the overexpression of the *Ae. aegypti* alpha subunit of ATP synthase at 48 hpi (Additional file 6: Table S4). In support of this observation, *Ae. albopictus* cell culture infected with CHIKV showed an increase in the beta subunit of ATP synthase expression [27].

### Replication complexes and the budding of new virions

Vesicle-associated membrane proteins (VAMPs) and an Aag-2 protein from the synaptobrevin family are among the most abundant proteins in 48 hpi (Additional file 6: Table S4). VAMPs are integral membrane components that mediate vesicle secretion [37]. In the process of new particle maturation, alphaviruses are assembled in vacuoles of the exocytic pathway in mosquito cells [38]. Jose et al. [35] have shown that alphavirus budding occurs on both the plasma membrane and internal vesicles. Thus, the budding of new viral particles by exocytic pathways may explain an increasing expression of VAMPs at 12 hpi and at 48 hpi. According to the growth kinetics of MAYV in Aag-2 cells, the highest production of infectious viral particles was observed at 48 hpi, corresponding to the peak abundance of VAMP.

### Nuclear transport

At 12 hpi, the host cell transcription factor C1 (HCFC1) was upregulated in *Ae. aegypti* cells. (Additional file 6: Table S4) In humans, HCFC1 is involved in the transcription of immediate herpes simplex virus genes [39]. In this context, human HCFC1 forms a protein complex with the viral DNA still in the cytoplasm mediated by the viral protein 16 (VP16) and directs it to the nucleus, commanding the expression of the immediate proteins [39]. According to Freiman and Herr [40], the association VP16-HCFC1 also occurs in invertebrates, suggesting that it is an evolutionarily conserved interaction. For example, even though herpes simplex virus has a DNA genome and nuclear replication, it was detected that RNA viruses with cytoplasmic replication infecting *Drosophila* also have a nuclear step [41]. Viral RNA was reverse-transcribed by the host cell and incorporated into genomic DNA as retrotransposon sequences to activate the RNAi pathway [41]. Thus, even in RNA viruses with cytoplasmic replication, it is possible that a nuclear step can be performed by the host cell as an antiviral mechanism. Although, as far as we know, no study has focused on this process involving HCFC1 during MAYV infection, this hypothesis should be investigated, given RNAi is the main antiviral mechanism in invertebrate cells.

## Conclusions

To better understand the mechanisms by which the Mayaro virus affects mosquito host cells, we performed a comparative proteomic analysis of MAYV-infected Aag-2 cells during the time-course at 12 hpi and 48 hpi. By mass spectrometry analysis, we identified 161 mosquito regulated proteins during MAYV infection. This proteome analysis of *Ae. aegypti* Aag-2 cells showed that differentially regulated proteins could be grouped into four clusters based on patterns of abundance change during the time course of infection. The results strongly suggest that the mosquito regulated proteins may be associated with the major infective events of MAYV in the host cell, from virus internalization, cell response to stress, energy demand, and new particle maturation. Some of our findings showed an increased expression of proteins such as prohibitin, synaptobrevin, transcription factor HCFC1, HSPs and enolase phosphatase e1, suggesting an important role of these proteins during MAYV infection. We also show that the glycolysis pathway was strongly modulated upon infection of insect cells indicating that infection is associated with a substantial alteration of host metabolism. Overall, this study provides a comprehensive understanding of the molecular basis of MAYV infection in *Ae. aegypti* Aag-2 cells by identifying several potential proteins and pathways directly related to the infection and consequent regulation of biochemical processes.

## Supplementary information

**Additional file 1: Figure S1.** Variable correlation plot for PCA demonstrating the quality of each variable represented by the distance of the arrow and the origin. The color intensity indicates the contribution of a variable to the components formation and the distance of each arrow from the origin indicates the quality of this feature.

**Additional file 2: Table S1.** Protein accession numbers of the 4 clusters identified through the VSClust algorithm corresponding to Fig. 3a.

**Additional file 3: Figure S2.** Estimation of optimal number of clusters according to the protein abundances over time. The optimal number is defined by the minimum centroid distance, indicated by the black square on the image. The optimal number was used to perform the clustering in Fig. 3a. Analysis performed in the VSClust online platform.

**Additional file 4: Table S2.** All peptides from the three different infection time points (0 as control, 12 hpi and 48 hpi), providing protein identification. Parameters used: FDR criterion of 1% and at least two unique peptides.

**Additional file 5: Table S3.***Aedes aegypti* Aag-2 cell and Mayaro virus proteins identified over the different infection time points.

**Additional file 6: Table S4.***Aedes aegypti* Aag-2 cell proteins with modulated abundance over the different infection time points and classification by GO terms for cellular component and biological process, obtained using the software Blast2Go corresponding to Fig. 5.

## Data Availability

Data supporting the conclusions of this article are included within the article and its additional files. Mass spectrometer output files (raw data) are available from the MassIVE database (accession number MSV000084687, 10.25345/c5h67w, https://massive.ucsd.edu/ProteoSAFe/dataset.jsp?task=da6985a8dcdd47b0aa0a8bc105c814c0) and ProteomeXchange (accession number PXD016737) [42–44].

## Abbreviations

ATP: Adenosine triphosphate
cDNA: Complementary DNA
CHIKV: Chikungunya virus
CPE: Cytopathic effects
DDA: Data-dependent acquisition
DENV: Dengue virus
DMEM: Dulbecco’s modified Eagle medium
Dpi: Day(s) post-infection
DTT: Dithiothreitol
EDTA: Ethylenediamine tetraacetic acid
ENOPH1: Enolase phosphatase e1
EPDA: End-point dilution assay
FBS: Fetal bovine serum
FDR: False discovery rate
GO: Gene Ontology
HCFC1: Host cell transcription factor C1
Hpi: Hour(s) post-infection
HSC: Heat shock cognate
HSP: Heat-shock protein
KEGG: Kyoto Encyclopedia of Genes and Genomes
LC-MS/MS: Liquid chromatography tandem mass spectrometry
MAYV: Mayaro virus
MOI: Multiplicity of infection
PCA: Principal components analysis
RISC: RNA-induced silencing complex
RNA: Ribonucleic acid
RNAi: RNA interference
RT-PCR: Reverse transcription polymerase chain reaction
VAMPs: Vesicle-associated membrane proteins
VP16: Viral protein 16
