# Supplementary figures and images for: Dynamic proteomic analysis of Aedes aegypti Aag-2 cells infected with Mayaro virus
Source: Parasit Vectors. 2020 Jun 10;13:297. doi: 10.1186/s13071-020-04167-2 (PMC7285477; doi:10.1186/s13071-020-04167-2)

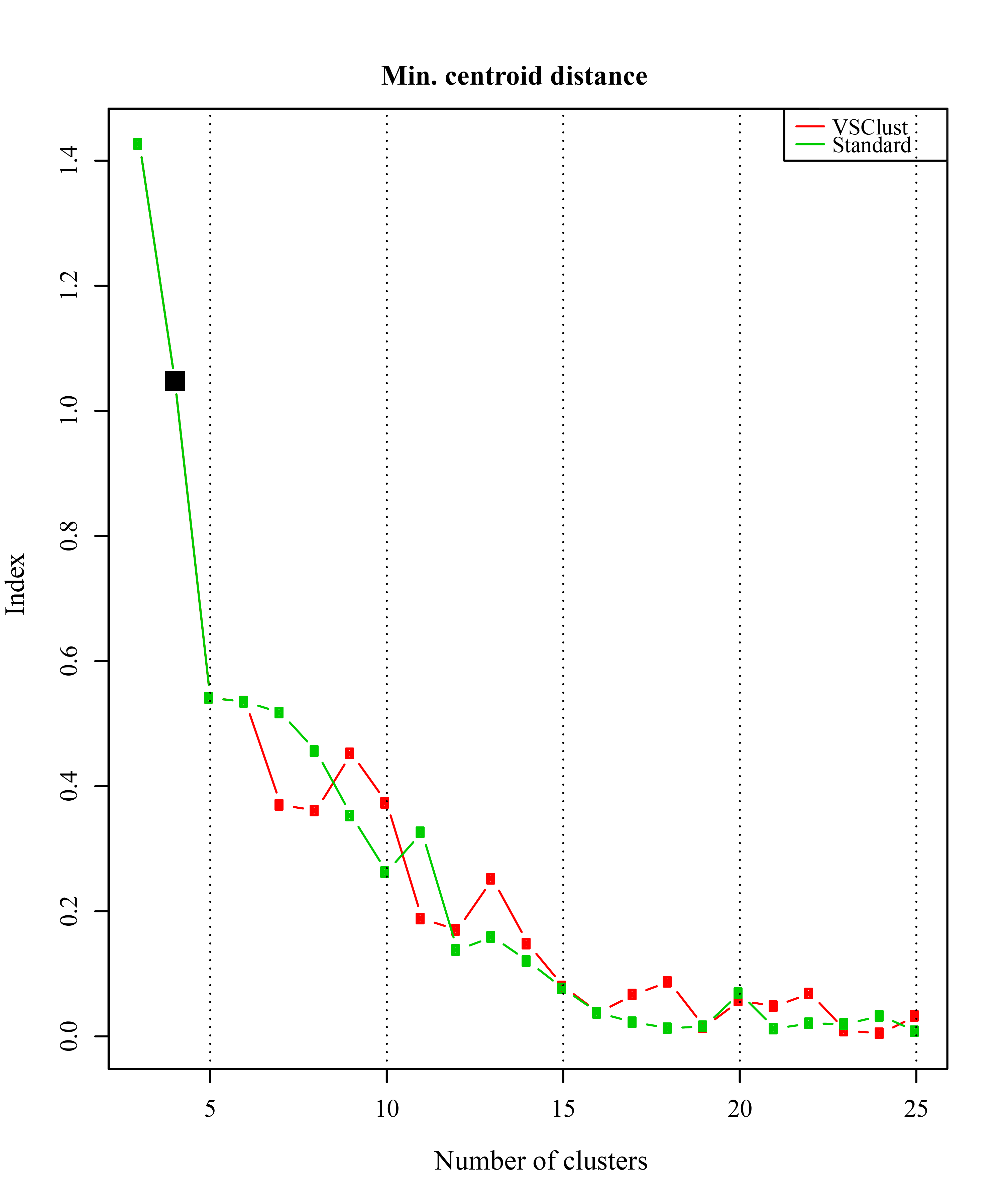

Supplement: Supplementary file 3 — Additional file 3: Figure S2. Estimation of optimal number of clusters according to the protein abundances over time. The optimal number is defined by the minimum centroid distance, indicated by the black square on the image. The optimal number was used to perform the clustering in Fig. 3a. Analysis performed in the VSClust online platform. [file 13071_2020_4167_MOESM3_ESM.tif]
